# Thromboseprophylaxe in Orthopädie und Traumatologie: Österreichischer Konsensus 2026

**DOI:** 10.1007/s00508-026-02781-1

**Published:** 2026-08-04

**Authors:** Michael Humenberger, Sibylle Langenecker, Anna Antoni, Cihan Ay, Herbert Bachler, Gabriella Cerna-Stadlmann, Thomas Feurstein, Alexander Haushofer, Florian Prüller, Eva Schaden, Fabian Schmid, Rene El Attal, Vinzenz Auersperg, ÖGAM Österreichische Gesellschaft für Allgemeinmedizin und Familienmedizin, Herbert Bachler, Thomas Feurstein, Eva Schaden, Cihan Ay, Thomas Gary, Fabian Schmid, Markus Theurl, Alexander Haushofer, Florian Prüller, Vinzenz Auersperg, Thomas Freude, Björn Rath, Anna Antoni, René El Attal, Gabriella Cerna-Stadlmann, Michael Humenberger, Sibylle Langenecker, ÖGARI Österreichische Gesellschaft für Anästhesiologie, Reanimation, Intensivmedizin, ÖGHO Österreichische Gesellschaft für Hämatologie & Medizinische Onkologie, ÖGIA Österreichische Gesellschaft für Internistische Angiologie, ÖGLMKC Österreichische Gesellschaft für Laboratoriumsmedizin und Klinische Chemie, ÖGO Österreichische Gesellschaft für Orthopädie, ÖGOuT Österreichische Gesellschaft für Orthopädie und Traumatologie, ÖGPMR Österreichische Gesellschaft für Physikalische Medizin und Rehabilitation, ÖGU Österreichische Gesellschaft für Unfallchirurgie

**Affiliations:** 1 Universitätsklinik für Orthopädie und Unfallchirurgie, Klinische Abteilung für Unfallchirurgie, Medizinische Universität Wien, Wien, Österreich; 2 Abteilung für Anästhesie und Intensivmedizin, Evangelisches Krankenhaus Wien, Wien, Österreich; 3 Universitätsklinik für Innere Medizin I. Klinische Abteilung für Hämatologie und Hämostaseologie, Medizinische Universität Wien, Wien, Österreich; 4 Niedergelassen, Innsbruck, Österreich; 5 Institut für Physikalische Medizin und Rehabilitation, Landeskrankenhaus Innsbruck, Innsbruck, Österreich; 6 Abteilung für Anästhesie und Intensivmedizin, Landeskrankenhaus Feldkirch, Feldkirch, Österreich; 7 Klinisches Institut für Medizinische und Chemische Labordiagnostik, Medizinische Universität Graz, Graz, Österreich; 8 Universitätsklinik für Anästhesie, Allgemeine Intensivmedizin und Schmerztherapie, Medizinische Universität Wien, Wien, Österreich; 9 Innere Medizin I, Landeskrankenhaus Feldkirch, Feldkirch, Österreich; 10 Orthopädie und Unfallchirurgie, Landeskrankenhaus Feldkirch, Feldkirch, Österreich; 11 Abteilung für Orthopädie und orthopädische Chirurgie, Pyhrn-Eisenwurzen Klinikum Kirchdorf und Steyr, Steyr, Österreich

**Keywords:** Thromboseprophylaxe, Hypokoagulation, Thromboembolie, Prophylaxe, Thrombosis prophylaxis, Hypocoagulation, Thromboembolism, Prophylaxis

## Abstract

Venöse Thromboembolien (VTE), eine Krankheitsentität die tiefe Venenthrombosen (TVT) und/oder Lungenembolien (PE) umfasst, gehören in der Orthopädie und Traumatologie zu den häufigsten und potenziell lebensbedrohlichen Komplikationen [1]. Die bislang gültige österreichische Empfehlung zur VTE-Prophylaxe bei muskuloskeletalen Eingriffen aus dem Jahr 2014 [2] bildet die aktuellen evidenzbasierten pharmakologischen und nichtpharmakologischen Strategien nicht mehr adäquat ab. Aktuelle internationale interdisziplinäre Leitlinien zur VTE-Prophylaxe wie von der European Society of Anaesthesiology and Intensive Care (ESAIC) [3] und der Arbeitsgemeinschaft Wissenschaftlich-medizinischer Fachgesellschaften (AWMF) [4] wurden daher unter Berücksichtigung österreichischer Versorgungsstrukturen adaptiert. Insbesondere fehlen bislang klar definierte Kriterien für ein funktionelles Frühmobilisationskonzept im Sinne eines Fast-Track-Programms sowie strukturierte Empfehlungen zur VTE-Prophylaxe bei konservativen orthopädisch-traumatologischen Behandlungen. Zur Erarbeitung praxisorientierter, evidenzbasierter Empfehlungen wurde eine interdisziplinäre Konsensusgruppe aus Vertreter:innen der für dieses Thema relevanten österreichischen Fachgesellschaften einberufen. Das Ziel war, strukturiertere, risikoadaptierte Handlungsempfehlungen unter Berücksichtigung der österreichischen Versorgungsrealität zu formulieren.

## Abstract (en)

Venous thromboembolism (VTE), a disease entity comprising deep vein thrombosis (DVT) and/or pulmonary embolism (PE), represents one of the most frequent and potentially life-threatening complications in orthopaedics and traumatology [1]. The previous Austrian recommendation on VTE prophylaxis in musculoskeletal procedures, dating from 2014 [2], no longer adequately reflects current evidence-based pharmacological and nonpharmacological strategies. Current international interdisciplinary guidelines on VTE prophylaxis, such as those issued by the European Society of Anaesthesiology and Intensive Care (ESAIC) [3] and the Association of the Scientific Medical Societies in Germany (AWMF) [4], were therefore adapted with consideration of Austrian healthcare structures. In particular, clearly defined criteria for a functional early mobilisation concept within the framework of a fast-track programme, as well as structured recommendations on VTE prophylaxis in conservative orthopaedic and traumatological treatments, have been lacking to date. To develop practice-oriented, evidence-based recommendations, an interdisciplinary consensus group comprising representatives of the Austrian medical societies relevant to this topic was convened. The aim was to formulate more structured, risk-adapted recommendations that take the Austrian healthcare context into account.

## Methodik

Die vorliegenden Empfehlungen wurden im Rahmen eines strukturierten interdisziplinären Konsensusprozesses erarbeitet. Thematische Schwerpunkte wurden von nominierten Expert:innen der Fachgesellschaften bearbeitet. Die Ausarbeitung erfolgte evidenzbasiert unter Berücksichtigung aktueller internationaler Leitlinien sowie der verfügbaren Primärliteratur.

Das konsolidierte Manuskript wurde in mehreren strukturierten Online-Konferenzen vorgestellt, diskutiert und in E‑Mail-Runden innerhalb der Expertengruppe finalisiert. Bei Inhalten mit weniger als 75 % Zustimmung im finalen Online-Expertentreffen war ein elektronisches Abstimmungsverfahren nach einem modifizierten Delphi-Prozess vorgesehen, mit Angabe der jeweiligen Konsensstärke im Text (in % Zustimmung). Ein starker Konsens wurde als > 95 % Zustimmung, ein Konsens als 75–95 %, eine mehrheitliche Zustimmung als 50–75 % Zustimmung und ein Dissens als < 50 % Zustimmung definiert.

Alle inhaltlichen Themen erreichten 100 % Zustimmung.

## Pathophysiologie

Die Entstehung von VTE lässt sich klassisch nach der Virchow-Trias beschreiben, die aus Endothelschädigung, venöser Stase und Hyperkoagulabilität besteht. Orthopädische und traumatologische Patient:innen sind häufig zugleich mehreren dieser Einflussfaktoren ausgesetzt. Endothelschädigungen treten im Rahmen chirurgischer Manipulationen, bei Frakturen oder nach traumatischen Gewebsverletzungen auf. Eine venöse Stase entsteht insbesondere durch Immobilisation, Gipsruhigstellung oder längere Bettlägerigkeit und führt zu einer Reduktion des venösen Rückstroms. Hinzu kommt eine trauma- und operationsbedingte Hyperkoagulabilität, die durch eine systemische Akut-Phase-Reaktion bzw. Entzündungsreaktionen sowie medikamentöse oder hormonelle Einflüsse zusätzlich verstärkt werden kann. Das Zusammenwirken dieser Faktoren erklärt das im muskuloskeletalen Setting erhöhte VTE-Risiko und begründet die zentrale Bedeutung präventiver Maßnahmen.

## Risikostratifizierung

Eine individualisierte Risikobewertung ist essenziell. Das Caprini Risk Assessment Model (RAM) ist ein weitverbreitetes Instrument zur Einschätzung des VTE-Risikos bei chirurgischen Patient:innen [5, 6]. Dies ist jedoch sehr aufwendig und nicht für orthopädisch-traumatologische Eingriffe validiert [7]. Insbesondere die risikoreduzierende Auswirkung der Frühmobilisation wird im Caprini-Score nicht berücksichtigt, weshalb er für das Scoring in der Fast-Track-Endoprothetik der unteren Extremität nicht herangezogen werden kann.

### Patientenspezifische VTE-Risikofaktoren

Die Anamneseerhebung kann folgende patientenabhängige Faktoren in einem vereinfachten Thrombose-Risiko-Check beinhalten (modifiziert nach ESAIC [8]):

- stattgehabte TVT und/oder PE (inklusive bei Blutsverwandten),
- Thrombophilie: z. B. Protein-C-Mangel, Protein-S-Mangel, AT-III-Mangel, Faktor-V-Leiden-Mutation, Prothrombin-Mutation, Antiphospholipidantikörpersyndrom,
- akutes Ereignis innerhalb des letzten Monats: Insult, Herzinfarkt, dekompensierte Herzinsuffizienz, immobilisierende Fraktur, Sepsis, große Operation,
- Erkrankungen: aktive Krebserkrankung (laufende Tumortherapie, Diagnose < 6 Monate, rezidivierende/metastasierende Erkrankung, palliative Situation),
- Adipositas BMI > 35 kg/m^2^,
- chronische Niereninsuffizienz (Kidney Disease Improving Global Outcomes, KDIGO-Klassifikation > G3a bzw. eGFR < 45 ml/min).

Trifft mindestens einer der genannten Punkte zu, ist von einem hohen individuellen VTE-Risiko auszugehen. Demgegenüber haben Patient:innen ohne einen der genannten Punkte ein niedriges patientenbezogenes VTE-Risiko.

Ein gesteigertes Risiko liegt vor [8], wenn zumindest ein Punkt aus folgender Liste zutrifft:

Alter > 75 Jahre, rheumatische Erkrankung, chronisch entzündliche Darmerkrankung, Herzinsuffizienz, chronisch obstruktive Lungenerkrankung, Schwangerschaft bis 6 Wochen nach der Entbindung.

### Eingriffsspezifische VTE-Risikofaktoren

Eingriffsspezifische Risikofaktoren tragen wesentlich zum thromboembolischen Gesamtrisiko bei und ergeben sich aus der Art, Dauer und dem Ausmaß des operativen oder traumatischen Geschehens. Ein erhöhtes VTE-Risiko besteht insbesondere bei großen orthopädischen und traumatologischen Eingriffen an der unteren Extremität, bei Becken- und Hüftchirurgie, bei Frakturen mit Immobilisation über 48 h sowie nach ausgedehnten Weichteil- oder Mehrfacheingriffen. Auch lange Operationszeiten (über 2 h), die Notwendigkeit von Folgeoperationen, die postoperative Ruhigstellung einer Extremität oder die eingeschränkte Belastbarkeit der unteren Extremität erhöhen das Risiko. In diesen klinischen Situationen ist von einem hohen VTE-Risiko auszugehen.

Demgegenüber sind kleine, kurz dauernde Eingriffe mit rascher Mobilisierung in der Regel mit einem deutlich niedrigeren eingriffsspezifischen VTE-Risiko verbunden [4].

Die Anwendung einer Blutsperre in der orthopädischen Chirurgie, insbesondere bei Knieendoprothesen, ist nach aktueller Evidenz nicht mit einem erhöhten Risiko klinisch relevanter venöser Thromboembolien assoziiert [9]. Zwar werden eine vorübergehende Hyperkoagulabilität sowie eine höhere Rate asymptomatischer distaler Unterschenkelthrombosen beschrieben, diese führen jedoch nicht zu einer Zunahme klinisch manifester VTE [10]. Daher kann die Blutsperre unter individueller Nutzen-Risiko-Abwägung eingesetzt werden.

### Blutungsrisiko

Das patientenbezogene und eingriffsspezifische Blutungsrisiko ist gegen das patientenbezogene und eingriffsspezifische VTE-Risiko abzuwägen, um die Indikation, Art, Dosis, Start und Dauer einer VTE-Prophylaxe festzulegen [11].

## Medikamentöse VTE-Prophylaxe

Grundsätzlich haben Ärzt:innen medikamentöse VTE-Prophylaxe auf Basis gültiger Leitlinien und anerkannter wissenschaftlicher Evidenz einzusetzen. Die zentralen Prinzipien medizinischer Entscheidungsfindung – nachgewiesene Wirksamkeit, ein günstiges Nutzen-Risiko-Verhältnis sowie das Fehlen gleichwertiger zugelassener Therapiealternativen – gelten gleichermaßen für den zulassungsüberschreitenden Einsatz von Arzneimitteln (Off-label-Use).

### Substanzen

#### Niedermolekulare Heparine (LMWH)

Als Standard der perioperativen VTE-Prophylaxe gelten LMWH. Sie zeichnen sich durch hohe Wirksamkeit und ein günstiges Sicherheitsprofil aus [4].

#### Direkte orale Antikoagulanzien (DOAK)

Aus der Gruppe der DOAK sind Apixaban, Rivaroxaban und Dabigatran für die Thromboseprophylaxe nach elektiven Knie- und Hüftendoprothesen zugelassen und in aktueller Praxis als Standardtherapie etabliert. Nachteil gegenüber LMWH ist eine tendenziell höhere Rate lokaler Hämatome [12]. In einer kürzlich publizierten Studie (PRONOMOS-Studie [13]) zeigte sich bei Patient:innen nach kleineren orthopädisch-traumatologischen Eingriffen der unteren Extremität eine niedrigere VTE-Rate bei der Gabe von Rivaroxaban im Vergleich zu Enoxaparin. Abseits der VTE-Prophylaxe nach Endoprothesenoperationen von Knie und Hüfte besteht derzeit bei orthopädisch-traumatologischen Behandlungen in Österreich keine Zulassung für DOAK.

#### Acetylsalicylsäure (ASS)

Früher ging man davon aus, dass ASS ausschließlich im arteriellen Gefäßsystem wirksam sei. Inzwischen ist belegt, dass ASS auch im venösen Schenkel Effektivität entfaltet. Für die medikamentöse VTE-Prophylaxe nach Hüft- oder Knieendoprothetik weisen LMWH sowie DOAK in der aktuellen S3-Leitlinie der AWMF ein Evidenzniveau 1a mit Empfehlungsgrad A auf. ASS wird bei geeigneten Patient:innen mit Empfehlungsgrad B empfohlen [4]. In den ESAIC Guidelines 2024 wird eine starke Empfehlung (Grad 1C) für ASS in der Kombination mit Fast-Track-Endoprothetik ausgesprochen [14].

Metaanalysen in 13 randomisierten klinischen Studien [15] sowie 21 nicht randomisierten Studien [16] bei Hüft- bzw. Kniegelenkersatzoperationen haben gegenüber anderen Antikoagulanzien hinsichtlich der VTE-Prophylaxe keinen Nachteil gezeigt. Studien zum sequenziellen Vorgehen – mit initialer Prophylaxe durch LMWH [17] oder Rivaroxaban-Prophylaxe [18] über 5 Tage und anschließender Umstellung auf ASS – belegten die Nichtunterlegenheit der ASS-Fortführung bei gleichzeitig geringerer Rate klinisch relevanter Blutungen. Eine aktuelle, multizentrische, retrospektive Studie [19] in einem großen Kollektiv von über 126.000 Patient:innen mit sowohl niedrigem als auch hohem VTE-Risiko identifizierte ASS als die wirksamste und sicherste Form der VTE-Prophylaxe nach Knieendoprothesenimplantation. Bei Patient:innen ohne patientenspezifische Risikofaktoren und Patient:innen im Fast-Track-Konzept sprechen, neben der Wirksamkeit, ein geringeres Risiko lokaler Hämatome, eine reduzierte Schwellneigung mit verbessertem Bewegungsumfang, ökonomische Vorteile und ein hoher Patientenkomfort für den Off-Label-Einsatz von ASS zur VTE-Prophylaxe [20, 21]. Die Evidenzlage ist jedoch heterogen. In der aktuellen CRISTAL-Studie mit über 9700 Patient:innen traten bei vergleichbaren Blutungskomplikationen mehr VTE-Ereignisse unter ASS auf (3,45 vs. 1,84 %), sodass die Nichtunterlegenheit gegenüber Enoxaparin nicht bestätigt werden konnte [22]. Neben methodischen Kritikpunkten [23] ist hierbei zu berücksichtigen, dass in der CRISTAL-Studie sowohl der Beginn der medikamentösen Prophylaxe als auch die nicht standardisierte Mobilisation erst am ersten postoperativen Tag erfolgten. Studien ohne dokumentiertes Fast-Track-Konzept – ebenso wie entsprechende Metaanalysen – sind vor diesem Hintergrund künftig nur eingeschränkt interpretierbar.

Aufgrund der uneinheitlichen Evidenzlage wird von Fachgesellschaften eine differenzierte Interpretation empfohlen [22–24]:

- Bei Immobilisation bzw. Mobilisation ohne Fast-Track ist LMWH gegenüber ASS überlegen. Eine generelle Substitution von LMWH durch ASS wird hier daher nicht empfohlen.
- Bei Patient:innen ohne patientenspezifische VTE-Risikofaktoren, Alter unter 75 Jahren, keine perioperative Fremdbluttransfusion, und ohne vorbestehende ASS-Therapie ist nach Hüft- bzw. Kniegelenkersatzoperation, bei Durchführung eines Fast-Track-Konzepts ASS empfohlen.

#### Pentasaccharid (Fondaparinux)

Fondaparinux stellt eine Alternative zur VTE-Prophylaxe dar. Es ist jedoch insbesondere bei älteren oder niereninsuffizienten Patient:innen mit einem erhöhten Blutungsrisiko assoziiert [4, 25].

#### Unfraktioniertes Heparin (UFH)

Unfraktioniertes Heparin hat in der Indikation der perioperativen VTE-Prophylaxe in Orthopädie und Traumatologie in Österreich keine Bedeutung mehr [26].

### Monitoring der medikamentösen VTE-Prophylaxe

Vor der Verordnung einer medikamentösen VTE-Prophylaxe sollen Serumkreatinin und die errechnete glomeruläre Filtrationsrate bestimmt werden [4].

Die labordiagnostische Überwachung der VTE-Prophylaxe ist bei orthopädischen oder traumatologischen Patient:innen in der Regel nicht erforderlich [4]. Dies gilt für LMWH, DOAK, ASS und Fondaparinux.

Bei Patient:innen mit Niereninsuffizienz kann eine Akkumulation von LMWH durch eine Überwachung der Anti-Xa-Aktivität (Talspiegel) erkannt werden.

Aufgrund des niedrigen Risikos des Auftretens von Heparin-assoziierten Komplikationen wie Osteoporose und Thrombozytopenie (HIT) im Rahmen der orthopädisch-traumatologischen medikamentösen VTE-Prophylaxe (bis 35 Tage) ist keine routinemäßige Überwachung des Knochenstatus und der Thrombozytenzahl notwendig. Bei hohem HIT-Risiko soll die Thrombozytenzahl bis zum 14. Tag 2- bis 3‑mal wöchentlich kontrolliert werden [4].

Bei Niereninsuffizienz oder Blutungsrisiko kann die Überwachung der DOAK durch die diluierte Thrombinzeit oder kalibrierte Spiegelbestimmungen erfolgen [27].

### Durchführung der medikamentösen VTE-Prophylaxe

Bei sofortiger Mobilisierung unter Vollbelastung ist keine VTE-Prophylaxe notwendig. Ist postoperativ eine entlastende Mobilisierung notwendig, sollte eine medikamentöse VTE-Prophylaxe verordnet werden. Bei Teilbelastung wird ebenfalls eine VTE-Prophylaxe empfohlen. Bei Patient:innen ohne sonstige VTE-Risikofaktoren kann hierbei individuell auf eine medikamentöse Prophylaxe verzichtet werden, sofern die Teilbelastung nur kurzzeitig notwendig ist und keine großen Gelenke ruhiggestellt werden. Als Beispiel ist hier die arthroskopische Teilmeniskektomie angeführt.

In der Vorfußchirurgie ist die medikamentöse VTE-Prophylaxe ebenfalls von der Mobilisierung nach dem Eingriff bzw. vorbestehenden VTE-Risikofaktoren abhängig. Ist eine Vollbelastung postoperativ möglich und bestehen keine patientenspezifischen Risikofaktoren, so ist keine medikamentöse VTE-Prophylaxe erforderlich [28].

Die Durchführung der medikamentösen VTE-Prophylaxe erfolgt risikoadaptiert (Blutungs- und VTE-Risiko) unter Berücksichtigung des Körpergewichts und der Nierenfunktion.

#### Gewichtsadaptierte Dosierung der VTE-Prophylaxe

Die medikamentöse VTE-Prophylaxe erfolgt in der Regel in Standarddosierung, z. B. Enoxaparin 4000 IE oder Dalteparin 5000 IE 1‑mal täglich subkutan. Eine routinemäßige Gewichtsadaption ist laut Leitlinie nicht vorgesehen, sollte jedoch bei extremen Gewichtssituationen erwogen werden [29]:

- Bei morbider Adipositas (BMI ≥ 35 kg/m^2^) oder hohem Körpergewicht (> 100 kg) besteht das Risiko unzureichender Wirkspiegel unter Standarddosierung. In diesen Fällen kann eine Dosiserhöhung von LMWH um 50 % (z. B. Enoxaparin 6000 IE) vorgenommen werden.
- Bei niedrigem Körpergewicht (< 50 kg oder BMI < 18,5 kg/m^2^) kann hingegen ein erhöhtes Blutungsrisiko bestehen. Hier kann die Dosierung halbiert werden.

Entscheidend ist keine starre Gewichtsgrenze, sondern die individuelle klinische Bewertung unter Berücksichtigung des Thrombose- und Blutungsrisikos. Die gewichtsabhängige Dosierung von DOAK ist bei orthopädisch-traumatologischen Patient:innen in der Regel nicht erforderlich [30–32].

Ebenso wurde in den verfügbaren Studien keine gewichtsadaptierte Dosierung von Low-Dose-ASS vorgenommen, und sie wird in den Leitlinien nicht empfohlen [33].

#### Anpassung bei Niereninsuffizienz

Da die Elimination von LMWH und DOAK überwiegend über die Nieren erfolgt, besteht bei Niereninsuffizienz aufgrund der Akkumulation ein erhöhtes Blutungsrisiko. Ab KDIGO-Klassifikation > G3a bzw. bei eGFR < 45 ml/min sollte eine Dosisreduktion erwogen werden, ggf. mit labordiagnostischer Überwachung.

#### Beginn der medikamentösen VTE-Prophylaxe

Die VTE-Prophylaxe sollte frühestmöglich begonnen werden, sobald eine stabile Hämostase im OP-Gebiet vorliegt. Dies ist im Regelfall 6–12 h postoperativ möglich.

#### Typische Anwendungsschemata in Österreich

- LMWH: z. B. Enoxaparin 4000 IE s.c. 1‑mal täglich, Beginn 6(–12) h postoperativ
  Synthetisches Pentasaccharid: Fondaparinux 2,5 mg s.c. 1‑mal täglich, Beginn frühestens 6 h postoperativ
- DOAK:
  - Apixaban 2,5 mg per os 2‑mal täglich, Beginn 12–24 h postoperativ
  - Rivaroxaban 10 mg per os 1‑mal täglich, Beginn (6–)10 h postoperativ
  - Dabigatran 220 mg (150 mg bei Alter ≥ 75 Jahre, Blutungsrisiko und/oder Niereninsuffizienz) per os 1‑mal täglich, Beginn 1–4 h postoperativ in halber Initialdosis
- ASS 100 mg per os 1‑mal täglich, Beginn 6(–12) h postoperativ

Die frühpostoperative Verordnung von initialem LMWH während des stationären Aufenthalts mit Umstieg auf perorale Weiterführung der VTE-Prophylaxe mit DOAK nach Krankenhausentlassung ist gängige Praxis und leitlinienkonform [29]. Der Umstieg erfolgt üblicherweise zum nächsten Verabreichungszeitpunkt, wobei eine Doppelantikoagulation zu vermeiden ist. Damit wird das Risiko von Blutungskomplikationen durch DOAK bei frischoperierten Patient:innen reduziert und eine etwaige Unsicherheit bei der gastrointestinalen Resorption der oral verfügbaren Medikamente, beispielsweise aufgrund von postoperativem Erbrechen, umgangen. Der ebenfalls gängige und leitliniengerechte Umstieg von initialem LMWH auf ASS hat ebenso den Hintergrund der Absicherung der Bioverfügbarkeit und Nutzung der medikamentösen VTE-Prophylaxe mit LMWH als Goldstandard im stationären Setting.

Die Dauer der medikamentösen VTE-Prophylaxe soll sich am Fortbestand relevanter Risikofaktoren orientieren. Obwohl die optimale Dauer der Thromboseprophylaxe nach orthopädischen Operationen nicht eindeutig untersucht ist, wird eine verlängerte Prophylaxe über 10 bis 14 Tage nach Kniegelenkersatzoperation sowie über 28 bis 35 Tage nach Hüftgelenkersatzoperation oder bis 35 Tage nach Kniegelenkersatzoperationen mit hohem patientenspezifischem VTE-Risiko oder nach einer Operation nach Hüftfraktur gängige Praxis und in den Guidelines empfohlen [29, 34]. Eine darüber hinaus verlängerte Verordnung sollte beispielsweise nach bilateraler Endoprothetik, bei VTE- bzw. PE-Anamnese oder bei stark eingeschränkter Mobilität bei der Entlassung erwogen werden.

Eine verkürzte Verordnung der VTE-Prophylaxe nach Hüft- und Kniegelenkersatz könnte unter bestimmten Bedingungen erwogen werden. Eine prospektive Kohortenstudie mit 17.582 Hüft- und Kniegelenkersatzoperationen unter Fast-Track zeigte bei einer ausschließlich während des Krankenhausaufenthalts (≤ 5 Tage) verordneten VTE-Prophylaxe eine niedrige Inzidenz symptomatischer VTE von nur 0,40 % [35]. Dies deutet darauf hin, dass eine verlängerte Thromboseprophylaxe möglicherweise Patient:innen mit verzögerter Mobilisation, bei längerem Krankenhausaufenthalt oder spezifischen Risikofaktoren vorbehalten bleiben kann. Darüber hinaus ergab eine Studie der Nordic Arthroplasty Register Association (NARA), dass es innerhalb von 90 Tagen nach einer Totalendoprothese der Hüfte keinen klinisch relevanten Unterschied im Risiko für VTE und größere Blutungen in Abhängigkeit von der Dauer der Thromboseprophylaxe gab [36]. Nach Knieendoprothetik kann die untere Dauergrenze von 10 Tagen ausreichend sein, wenn ein sehr niedriges VTE-Risiko vorliegt (rasche und vollständige Mobilisation, keine individuellen VTE-Risikofaktoren, komplikationsloser Verlauf) – dies soll laut AWMF-Empfehlung jedoch eine Einzelfallentscheidung sein und keine pauschale Verkürzung [4].

Arthroskopien des Knie- oder Hüftgelenks erfordern bei fehlenden Risikofaktoren keine routinemäßige medikamentöse Prophylaxe, sofern keine postoperative Immobilisation erfolgt und die Tourniquet-Zeiten < 60 min bleiben [24, 37]. Ist postoperativ keine Vollbelastung möglich, wird für 7 bis 10 Tage bzw. bis zur sicheren Mobilisation eine Prophylaxe mit LMWH empfohlen [4].

## Nichtmedikamentöse (mechanische) VTE-Prophylaxe

Die nichtmedikamentöse VTE-Prophylaxe, zu der die Frühmobilisation als Basismaßnahme sowie mechanische Methoden gezählt werden, soll die intravasale Strömungsgeschwindigkeit erhöhen und damit dem Faktor Stase in der Virchow-Trias entgegenwirken.

### Medizinische Thromboseprophylaxestrümpfe (MTPS)

Der Stellenwert der additiven MTPS bei indizierter medikamentöser Prophylaxe wird in den letzten Jahren infrage gestellt. Während in der letzten österreichischen Leitlinie 2007 zu diesem Thema das Tragen von MTPS zusätzlich zur medikamentösen Prophylaxe noch deutlich empfohlen wurde und in der deutschen Leitlinie von 2015 nach Expertenkonsens, v. a. bei Kontraindikationen gegen die medikamentöse Prophylaxe, bei den allermeisten Patientenkollektiven eine optionale Empfehlung ausgesprochen wurde, scheint dies nach aktueller Studienlage nicht mehr notwendig zu sein. Damit können sowohl der Kostenfaktor als auch der Aufwand für die Patient:innen minimiert werden [2, 24].

Cohen et al. konnten in einer 2007 veröffentlichten randomisierten, einfach verblindeten Studie mit über 800 Patient:innen nach Hüftoperationen zeigen, dass mit dem Einsatz von MTPS zusätzlich zur medikamentösen Prophylaxe kein signifikanter Nutzen bei der Prävention von VTE-Ereignissen erzielt wird [38]. Shalhoub et al. konnten in einer randomisierten, kontrollierten, multizentrischen Studie mit über 1800 Patient:innen (GAPS-Studie) das Fehlen eines signifikanten Unterschieds zwischen Patient:innen mit alleiniger medikamentöser Prophylaxe und der Kombination aus medikamentöser und MTPS-Prophylaxe über die Dauer von 90 Tagen postoperativ hinsichtlich TVT und PE zeigen [39]. Auch eine große Metaanalyse von Turner et al. 2024 kommt zu dem Schluss, dass die Erweiterung der medikamentösen Thromboseprophylaxe durch MTPS nicht notwendig ist [40]. Hinsichtlich des Typs der MTPS wurde 2012 eine Cochrane-Analyse zum Vergleich von knie- und oberschenkellangen MTPS durchgeführt. Diese schätzt die Studienlage zwar als schwach ein, die analysierten Studien zeigten jedoch keinen Unterschied bei den Thromboseereignissen zwischen den beiden MTPS-Arten [41]. Bei der Verordnung spielen daher Faktoren wie Kosten und Praktikabilität eine Rolle. Bezüglich der Wirksamkeit von MTPS bei Patient:innen mit geringem VTE-Risiko ohne Indikation zur medikamentösen Prophylaxe gibt es derzeit wenige aussagekräftige Daten. Dort wird vermutlich der PETS-Trial (Studienlaufzeit April 2022 bis Dezember 2025 – mit Stichtag 03/2026 noch keine Ergebnisse verfügbar) in Zukunft für Evidenz sorgen und eine punktuelle Anpassung der Leitlinien mit sich bringen. Diese groß angelegte Studie untersucht bei über 20.000 Patient:innen mit niedrigem VTE-Risiko den Effekt von MTPS im Vergleich zu keiner mechanischen Thromboseprophylaxe [42].

### Intermittierende pneumatische Kompression (IPK)

Sowohl die Datenlage als auch die Studienergebnisse sprechen für einen großen Wirksamkeitsvorteil der IPK gegenüber MTPS. Eine Metaanalyse 2013 mit über 16.000 berücksichtigten Patient:innen, davon bezogen sich 27 Studien auf orthopädisch-traumatologische Patient:innen, zeigte ein signifikant geringeres VTE-Risiko für Patient:innen mit IPK [43]. Die interdisziplinären Guidelines ordnen die IPK als wirksame mechanische Option ein [4, 33]. Der Einsatz der IPK gilt generell als sicher, wird gut toleriert und weist nur wenige potenzielle Komplikationen auf. Als Kontraindikationen sind v. a. eine dekompensierte Herzinsuffizienz, akute TVT oder eine schwere periphere arterielle Verschlusskrankheit zu beachten [44].

#### IPK bei Kontraindikation gegen medikamentöse VTE-Prophylaxe

Die deutsche S3-Leitlinie empfiehlt bei Patient:innen mit mittlerem oder hohem VTE-Risiko, bei denen die medikamentöse Prophylaxe kontraindiziert ist (z. B. wegen aktiver Blutung), als physikalische Maßnahme bevorzugt die IPK [4].

#### IPK in Kombination mit medikamentöser VTE-Prophylaxe

Einige Studien haben die Kombination aus medikamentöser Prophylaxe und IPK bei Patient:innen mit hohem VTE-Risiko untersucht, wobei die Ergebnisse hier nicht eindeutig sind [45, 46]. Laut den aktuellen Leitlinien der ESAIC [33] kann bei Patient:innen mit hohem Thromboserisiko die IPK zusätzlich zur pharmakologischen Prophylaxe erwogen werden.

#### Durchführung

Es gibt unterschiedliche Größen, Manschettenlängen (Fuß, Unterschenkel vs. Ganzbein), Kammeranzahlen (ein- vs. mehrkammerig) und Druckprofile (simultan vs. sequenziell/peristaltisch). Die Wahl der IPK sollte indikationsgerecht erfolgen. Bei hohem VTE-Risiko sind für die postoperative Prophylaxe Ganzbeinmodelle sinnvoll. Die Anlage der IPK kann je nach operativem Eingriff und der Zugänglichkeit der unteren Extremität bereits intraoperativ bzw. unmittelbar postoperativ erfolgen. Die Anwendung erfolgt möglichst kontinuierlich mit Unterbrechungen für Pflege und Mobilisation, solange ein relevantes VTE-Risiko besteht bzw. bis die medikamentöse VTE-Prophylaxe verordnet werden kann.

### Frühmobilisation

Die frühzeitige Mobilisation ist durch die Aktivierung der Venenpumpe die wirksamste nichtmedikamentöse Maßnahme zur VTE-Prophylaxe [4]. Sofern nach der Operation bzw. im Rahmen der konservativen Behandlung voll- oder teilbelastet werden darf, soll tatsächlich mobilisiert werden. Als Voraussetzungen gelten postoperativ die hämodynamische Stabilität, die ausreichende Vigilanz, eine nebenwirkungsarme Schmerzkontrolle, die Patientenedukation sowie die bestehende Compliance. Im systematischen Review von Lau et al. wurde dargestellt, dass Frühmobilisation in vielen Fällen nicht als alleinige Maßnahme zur VTE-Prophylaxe ausreicht [47]. Guidelines empfehlen die Frühmobilisation spätestens ab 12–24 h postoperativ zusätzlich zur medikamentösen VTE-Prophylaxe, sofern indiziert [4, 33].

### Fast-Track-Konzept

Das Fast-Track-Konzept ist ein strukturiertes, multidisziplinäres und multimodales Behandlungskonzept, das darauf abzielt, die funktionelle Erholung zu beschleunigen. Dies führt zu einer Reduktion perioperativer Komplikationen und zur Verkürzung des Krankenhausaufenthalts. Das Konzept basiert auf der Kombination evidenzbasierter Maßnahmen entlang des gesamten Behandlungspfades. Es umfasst die präoperative Patientenedukation und Vorbereitung, optimierte Anästhesie- und Schmerzstrategien inklusive lokaler Infiltrationsanalgesie (LIA), muskelschonende OP-Techniken ohne mobilisationshemmende Drainagen bis hin zur frühpostoperativen Mobilisation und zu standardisierten funktionellen Entlassungskriterien.

Im Kontext der Thromboseprophylaxe verändert das Fast-Track-Konzept die Risikokonstellation wesentlich. Der frühzeitige Mobilisationsbeginn und die Reduktion immobilisierender Faktoren senken das Stase-bedingte Thromboserisiko. Eine pauschale Gleichsetzung großer orthopädischer Operationen mit einem hohen VTE-Risiko ist in diesem Setting daher nicht mehr adäquat [35]. Patientenbezogene Risikofaktoren sind jedoch weiterhin wirksam und bei der Verordnung der VTE-Prophylaxe zu berücksichtigen. Eine individuelle, mobilitäts- und risikoadaptierte Prophylaxestrategie rückt in den Vordergrund.

#### Durchführung

Aufgrund des geringeren VTE-Risikos sollten Fast-Track-Protokolle dem konventionellen Vorgehen bei Knie- oder Hüftendoprothetik vorgezogen werden. Wenn abgestufte Strategien der medikamentösen VTE-Prophylaxe nach Endoprothesenoperationen der unteren Extremität im Rahmen des Fast-Track-Konzepts verordnet werden, sollten die Mindesttherapieziele der Frühmobilisation überprüft werden. Leider sind in den Guidelines keine konkreten numerischen Zielvorgaben definiert, z. B. hinsichtlich der Gehstrecke. Wichtiger als die Distanz dürfte das sichere Gangbild sein. In der klinischen Praxis haben sich folgende Ziele etabliert:

- Vertikalisierung und erste Schritte im Aufwachbereich (spätestens < 6[–12] h postoperativ),
- Gehstrecke 30 m mit sicherem Gangbild am 1. postoperativen Tag,
- 100 m und Treppenfähigkeit (1 Stockwerk) bis zur Entlassung.

Insgesamt setzt das Fast-Track-Konzept einen guten präoperativen funktionellen Status ohne ausgeprägte Gebrechlichkeit (Frailty) voraus. Zur objektiven Einschätzung können Gebrechlichkeitsskalen herangezogen werden, etwa die Clinical Frailty Scale mit einem Grenzwert von < 5 oder die Fried-Skala mit einem Grenzwert von ≤ 2 [48]. Obwohl gebrechliche Patient:innen von einer Mobilisation profitieren und damit Komplikationen vermieden werden, können ggf. die Zielkriterien von Fast-Track physisch nicht erfüllt werden.

Es erscheint hinsichtlich der VTE-Risikoreduktion nicht ausreichend, wenn nur 1‑mal täglich mobilisiert wird. Die Venenpumpe soll auch im Liegen und im Sitzen regelmäßig aktiviert werden. Patienteninformationsbroschüren mit Abbildungen geeigneter Übungen sowie Anwendungen für Smartphones mit Erinnerungsfunktionen für das Üben können krankenhausinterne Fast-Track-Protokolle unterstützen:

- Eigentraining mehrmals täglich im Liegen (z. B. Bein aufstellen, Vorfuß hochziehen), im Sitzen (z. B. Zehen‑/Fersenstand im Wechsel), im Stehen (z. B. Knie heben, Ferse zum Gesäß heben).

Das Ermutigen zum Benützen des neuen Gelenkimplantats kann auch z. B. durch das Servieren des Essens bei Tisch statt im Bett erfolgen. Das unterstreicht den multiprofessionellen Rahmen eines Fast-Track-Konzeptes. Die Schmerzkontrolle mit Zielwerten von ≤ 5 auf der 10-stelligen visuellen Analogskala (VAS) bei Bewegung ohne Nebenwirkungen wie Benommenheit, Gangunsicherheit oder motorische Blockade ist Voraussetzung für Fast-Track. Schmerz ist subjektiv und individuell zu therapieren. Dies erfordert Aufmerksamkeit und die Verabreichung von verordneten Analgetika bei Bedarf (z. B. VAS > 3) durch das Pflegepersonal, dessen Rolle für ein Fast-Track-Konzept erfolgsrelevant ist. Gleichzeitig spart die zunehmende Selbstständigkeit der Patient:innen unter Fast-Track durch das Vermeiden von Kathetern und Drainagen Pflegeaufwand.

#### Medikamentöse VTE-Prophylaxe nach Hüft- und Knieendoprothetik im Fast-Track-Konzept

In dieser Indikation gibt es in den Europäischen Guidelines zur Kombination mit Fast-Track starke Empfehlungen: LMWH (GRADE 1B), DOAK (GRADE 1B) oder ASS [14] (GRADE 1C). Während LMWH und DOAK auch ohne Kombination mit Fast-Track stark empfohlen werden, ist für ASS zur VTE-Prophylaxe nach Knie- oder Hüftendoprothetik die Kombination mit einer sorgfältigen Fast-Track-Behandlung erforderlich [14].

**Abb. 1:**
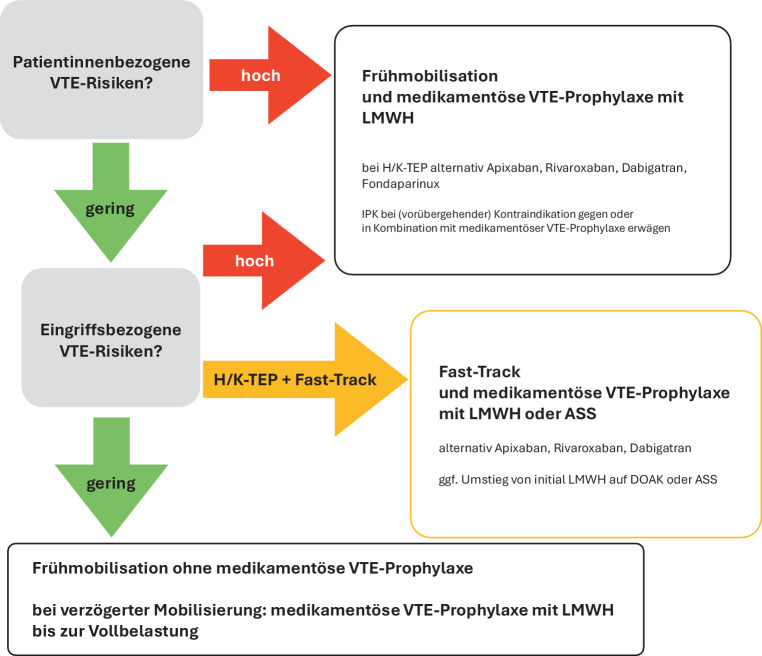
Entscheidungspfad zu Fast-Track mit medikamentöser VTE-Prophylaxe oder Frühmobilisation mit oder ohne medikamentöse VTE-Prophylaxe anhand der Stratifizierung patienten- und eingriffsbezogener VTE-Risiken

In Abb. 1 ist der Entscheidungspfad zu Fast-Track mit medikamentöser VTE-Prophylaxe oder Frühmobilisation mit oder ohne medikamentöse VTE-Prophylaxe anhand der Stratifizierung patienten- und eingriffsbezogener VTE-Risiken dargestellt.

### Vena-cava-inferior-Filter

Der Einsatz von Vena-cava-inferior-Filtern zur primären VTE-Prophylaxe ist nicht empfohlen.

## Spezielle Patient:innengruppen

### Hüftgelenknahe Frakturen

Hüftgelenknahe Frakturen weisen im Vergleich zum elektiven Hüftgelenkersatz ein erhöhtes VTE-Risiko auf [49]. Daher wird unabhängig von der Operationsmethode eine medikamentöse VTE-Prophylaxe für 28 bis 35 Tage empfohlen. Die Prophylaxe soll postoperativ begonnen werden, sobald eine stabile Hämostase gegeben ist, und kann bei persistierenden Risikofaktoren im Einzelfall verlängert werden [24]. Da DOAK nach Frakturen in Österreich nicht zugelassen sind und robuste Studiendaten fehlen, stellen LMWH bei Patient:innen nach hüftgelenknahen Frakturen die Mittel der Wahl dar. Die Bedeutung der frühestmöglichen Mobilisierung muss bei diesen meist geriatrischen Patient:innen besonders betont werden.

### Konservative Therapie (z. B. Gipsruhigstellung)

Die Ruhigstellung der unteren Extremität durch Gipsverbände oder immobilisierende Orthesen ist mit einer relevanten Mobilitätseinschränkung und einem erhöhten Risiko für venöse Thromboembolien assoziiert [50]. Betroffene Patient:innen werden je nach zusätzlichen patientenbezogenen Risikofaktoren und Immobilisationsdauer einem geringen bis höheren Risikoprofil zugeordnet. Aktuelle randomisierte Studien zeigen, dass eine routinemäßige pharmakologische VTE-Prophylaxe bei Patient:innen ohne individuelle Risikofaktoren nicht zwingend erforderlich ist, da in unselektierten Kollektiven kein signifikanter Vorteil hinsichtlich symptomatischer VTE nachgewiesen werden konnte [51]. Diese Evidenz hat bislang jedoch keinen vollständigen Niederschlag in internationalen Leitlinien gefunden. Die aktualisierte AWMF-S3-Leitlinie führt hierzu aus, dass Patient:innen mit fixierenden Verbänden – d. h. immobilisierenden Hartverbänden oder gleich wirkenden Orthesen an der unteren Extremität – und mittlerem oder hohem VTE-Risiko eine medikamentöse VTE-Prophylaxe empfohlen werden soll [4].

Vor diesem Hintergrund empfehlen wir folgendes pragmatisches Vorgehen:

- Bei Ruhigstellung eines großen Gelenks der unteren Extremität (Knie- oder Sprunggelenk) mittels eines Gipsverbands und (Teil‑)Entlastung sollte weiterhin eine medikamentöse Thromboseprophylaxe durchgeführt werden.
- Bei Ruhigstellung des Kniegelenks sollte auch bei Vollbelastung eine medikamentöse VTE-Prophylaxe durchgeführt werden. Gleiches gilt unabhängig von der Art der Immobilisation, wenn individuelle patientenbezogene Risikofaktoren für eine VTE vorliegen.
- Bei Versorgung mit einer funktionellen Knie- oder Sprunggelenkorthese oder einem Unterschenkelgips bei Vollbelastung kann hingegen auf eine medikamentöse Prophylaxe verzichtet werden, sofern keine individuellen Risikofaktoren vorliegen [25].

Zur Identifikation von Risikopatient:innen kann ein validiertes Risikostratifizierungsmodell, insbesondere der TRiP(cast)-Score, eingesetzt werden. Bei einem Score < 7 ist das Risiko einer symptomatischen VTE innerhalb von 3 Monaten niedrig (< 1 %), sodass in dieser Gruppe auf eine medikamentöse VTE-Prophylaxe verzichtet werden kann. Patient:innen mit einem Score ≥ 7 weisen hingegen ein erhöhtes Risiko auf und sollten eine pharmakologische Prophylaxe erhalten [52].

Als Standardmedikation wird ein LMWH in prophylaktischer Dosierung empfohlen, wobei auch ASS als Alternative erwogen werden kann [53]. Die Dauer der Prophylaxe orientiert sich am individuellen Risiko und an der Mobilitätssituation und wird in der Regel bis zur Beendigung der relevanten Immobilisation bzw. bis zum Erreichen einer sicheren Vollbelastung fortgeführt.

#### Polytrauma

Polytraumatisierte Patient:innen weisen aufgrund der Kombination aus schwerem Gewebetrauma, systemischer Inflammation, Immobilisation und häufig prolongierten Intensivaufenthalten ein deutlich erhöhtes Risiko für VTE auf. Detaillierte Empfehlungen zur VTE-Prophylaxe nach Polytrauma gehen über die Zielsetzung dieses Konsensus hinaus. Kurz zusammengefasst ist ein stufenweises, interdisziplinär abgestimmtes Vorgehen erforderlich: Bei initial noch bestehender Blutungsgefahr erfolgt der Beginn mit mechanischen Maßnahmen, gefolgt von der frühestmöglichen Einleitung der medikamentösen VTE-Prophylaxe [4]. Auch im Polytrauma-Setting kommt der frühmobilisationsorientierten Behandlung im Sinne des Konzepts der Early Appropriate Care mit täglicher Reevaluierung der Operabilität ein hoher Stellenwert zu [30, 54].

Bei gleichzeitigem Vorliegen eines Schädel-Hirn-Traumas stellt die Nutzen-Risiko-Abwägung für die medikamentöse VTE-Prophylaxe eine besondere Herausforderung dar. Lebensbedrohliche Nachblutungen müssen hierbei gegen das Risiko einer VTE abgewogen werden. Für Patient:innen mit vorbestehender antikoagulatorischer Therapie verweisen wir hierfür auf das diesbezügliche „Austrian interdisciplinary consensus statement“ [55].

### Patient:innen mit vorbestehender gerinnungshemmender Therapie

Falls bei Patient:innen mit bestehender antithrombotischer Medikation eine präoperative Therapiepause notwendig ist, sollte deren Dauer an die Indikation, das Thromboembolierisiko der Grunderkrankung, die Blutungsrisiken der geplanten Operation sowie die Nierenfunktion angepasst werden. Für Planung und Entscheidungsfindung gelten die von der ÖGARI herausgegebenen Leitlinien zum präoperativen Medikamentenmanagement auch für Eingriffe in der Orthopädie und Unfallchirurgie. Die Bestimmung geeigneter Pause- und Wiederaufnahmeintervalle kann evidenzbasiert, unter Berücksichtigung patient:innen- und eingriffsspezifischer Parameter, mithilfe der ÖGARI-Prämed-App unterstützt werden [56].

Ein Bridging von Vitamin-K-Antagonisten oder DOAK mit LMWH ist grundsätzlich nicht empfohlen, sondern nur in ausgewählten Einzelfällen indiziert und sollte Patient:innen mit besonders hohem thromboembolischem Risiko, wie z. B. einer mechanischen Herzklappe, vorbehalten bleiben [57]. Nach dem Eingriff erfolgt die Wiederaufnahme der vorbestehenden antithrombotischen Therapie, sobald eine stabile Hämostase vorliegt. Die zwischenzeitliche postoperative medikamentöse VTE-Prophylaxe mit LMWH wird in diesem Zusammenhang in der Regel beendet und in die orale Dauertherapie überführt.

Eine Sekundärprophylaxe mit ASS oder P2Y12-Inhibitoren kann postoperativ zusätzlich zur VTE-Prophylaxe mit LMWH fortgeführt werden.

### Operationen und Verletzungen der oberen Extremität

Nach Operationen oder Verletzungen an Gelenken, Knochen und Weichteilen der oberen Extremität sollen das individuelle VTE-Risiko sowie das Blutungsrisiko abgeschätzt werden. Wenn keine VTE-Risikofaktoren vorliegen, kann nach Operationen an der oberen Extremität auf eine medikamentöse VTE-Prophylaxe verzichtet werden.

Bei Patient:innen mit erhöhtem VTE-Risiko werden in der Regel eine Frühmobilisation sowie eine postoperative medikamentöse Thromboseprophylaxe empfohlen.

Die Dauer der Prophylaxe richtet sich nicht pauschal nach der Operationsart, sondern nach dem Fortbestehen relevanter Risikofaktoren (z. B. Immobilisation, zusätzliche dispositionelle Faktoren) und kann bei Bedarf über den stationären Aufenthalt hinaus fortgeführt werden.

Bestehen Kontraindikationen gegen eine medikamentöse VTE-Prophylaxe, sollen nichtpharmakologische Verfahren bevorzugt eingesetzt werden.

## Patient:inneninformation

Individuelle Risikofaktoren für VTE und Blutungen werden im österreichischen Versorgungskontext in der Regel im Rahmen der präanästhesiologischen Evaluierung systematisch erhoben. In besonderen Konstellationen – etwa bei hereditären Blutungsneigungen oder komplexen internistischen Vorerkrankungen – sollte die perioperative Strategie der VTE-Prophylaxe interdisziplinär, insbesondere unter internistischer Mitbeurteilung, festgelegt werden.

Die sich individuell daraus ergebende Strategie der VTE-Prophylaxe für die jeweils anstehende Operation soll im Rahmen des „informed consent“ mit den Patient:innen hinsichtlich Nutzen, Risiko und Alternativen besprochen werden. Ziel ist es, eine informierte Entscheidung zu ermöglichen und zugleich die Adhärenz zur verordneten VTE-Prophylaxe – auch über den stationären Aufenthalt hinaus – zu fördern.

## Fazit und Ausblick

**Abb. 2:**
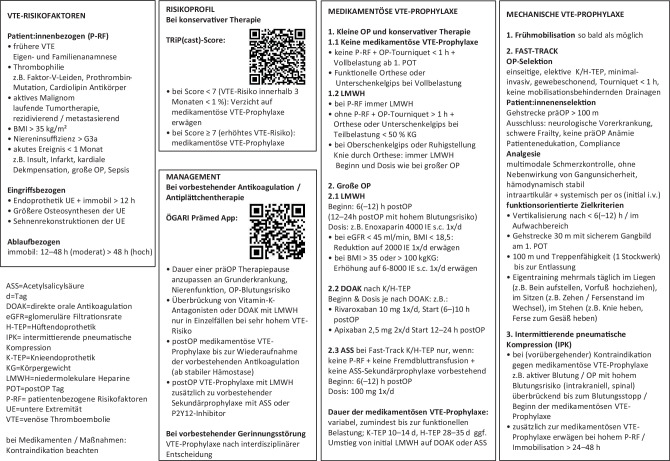
Kurzleitfaden der Thromboseprophylaxe in Orthopädie und Traumatologie nach Österreichischem Konsensus 2026

Die VTE-Prophylaxe ist ein integraler Bestandteil der Patientensicherheit in der Orthopädie und Unfallchirurgie. Eine risikoadaptierte, evidenzbasierte und zugleich pragmatische Strategie ist erforderlich, um das Thrombose- und Blutungsrisiko angemessen auszubalancieren. Der vorliegende Konsensus umfasst medikamentöse und mechanische Maßnahmen sowohl im operativen als auch im konservativen Setting. Zentrale Prinzipien sind die individuelle Risiko-Nutzen-Abwägung, die patientenzentrierte Umsetzung, die Integration von Fast-Track-Konzepten sowie die Harmonisierung klinischer Standards. In Abb. 2 ist ein Kurzleitfaden der Thromboseprophylaxe in Orthopädie und Traumatologie nach Österreichischem Konsensus 2026 dargestellt.

Faktor-XI-Inhibitoren stellen eine neue Klasse von Antikoagulanzien zur Thromboseprophylaxe dar. Derzeit (Stand 2026) sind FXI-Inhibitoren noch nicht zugelassen. Ergebnisse aus Phase-3-Studien stehen derzeit noch aus.

Zukünftig können digitale Risikostratifizierungsinstrumente, standardisierte Fast-Track-Protokolle sowie gesundheitsökonomische Analysen zur weiteren Optimierung beitragen. Ziel bleibt eine individualisierte, strukturierte und qualitätsgesicherte Thromboseprävention im österreichischen Versorgungskontext.

## Data Availability

Alle dieser Arbeit zugrunde liegenden Daten sind in diesem Artikel enthalten.
